# Supplementary material for: Alternative Healthy Eating Index-2010 and Incident Non-Communicable Diseases: Findings from a 15-Year Follow Up of Women from the 1973–78 Cohort of the Australian Longitudinal Study on Women’s Health
Source: Nutrients. 2022 Oct 20;14(20):4403. doi: 10.3390/nu14204403 (PMC9611717; doi:10.3390/nu14204403)
Supplement: Supplementary file 1 [file nutrients-14-04403-s001.zip › nutrients-1904378-supplementary.pdf]

Alternative Healthy Eating Index-2010 and incident non-communicable diseases: Findings from a 15-year follow up of women from the 1973-78 cohort of the Australian Longitudinal Study on Women's Health

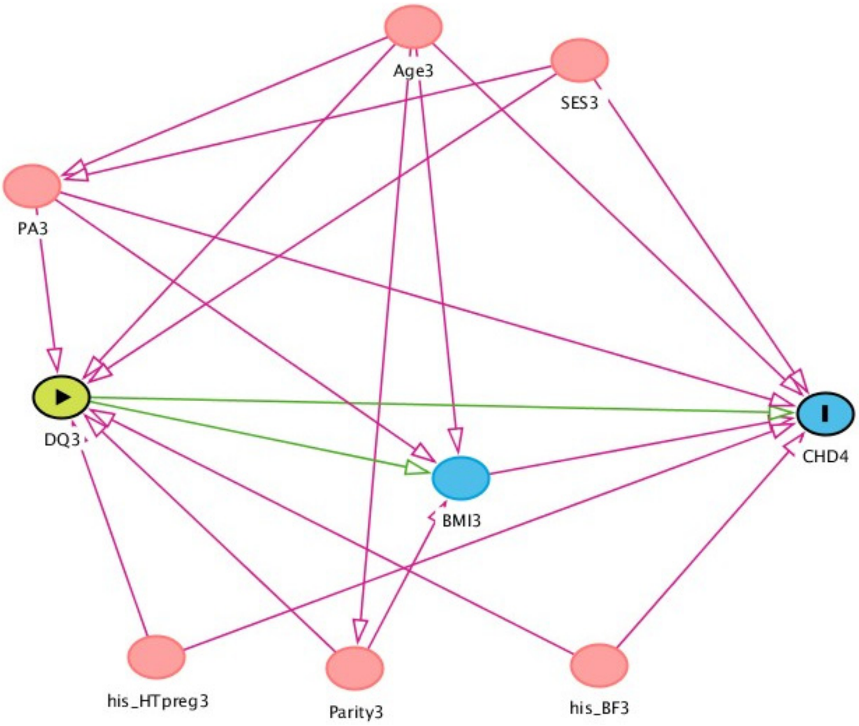

Supplementary Figure S1: A Directed Acyclic Graph (DAG) used to identify potential confounders for coronary heart disease at survey 4

BMI-Body mass index; CHD-coronary heart disease; DQ-diet quality; his_BF-history of breastfeeding; his_HTpreg-history of hypertension during pregnancy; PA-physical activity; SES-socioeconomic status (residence, marital status, education, occupation, income stress)

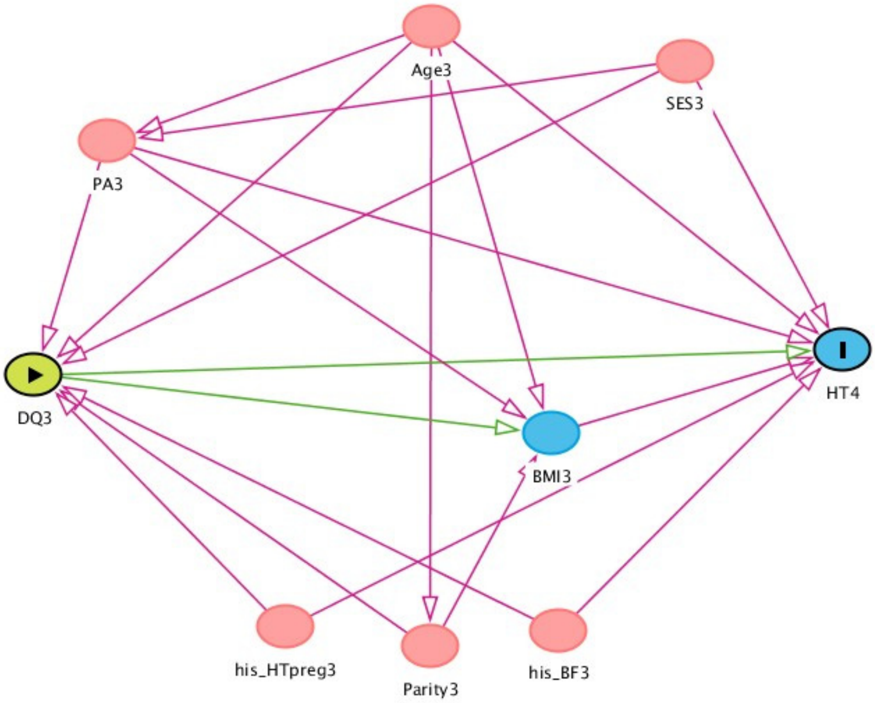

Supplementary Figure S2: A Directed Acyclic Graph (DAG) used to identify potential confounders for hypertension at survey 4

BMI-Body mass index; DQ-diet quality; his_BF-history of breastfeeding; his_HTpreg-history of hypertension during pregnancy; HT-hypertension; PA-physical activity; SES-socioeconomic status (residence, marital status, education, occupation, income stress)

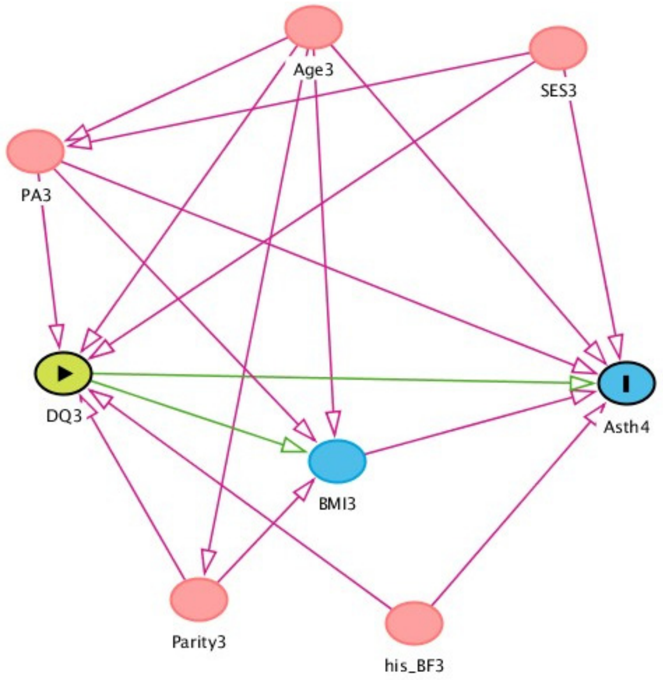

Supplementary Figure S3: A Directed Acyclic Graph (DAG) used to identify potential confounders for asthma at survey 4

Asth-asthma; BMI-Body mass index; DQ-diet quality; his_BF-history of breastfeeding; PA-physical activity; SES-socioeconomic status (residence, marital status, education, occupation, income stress)

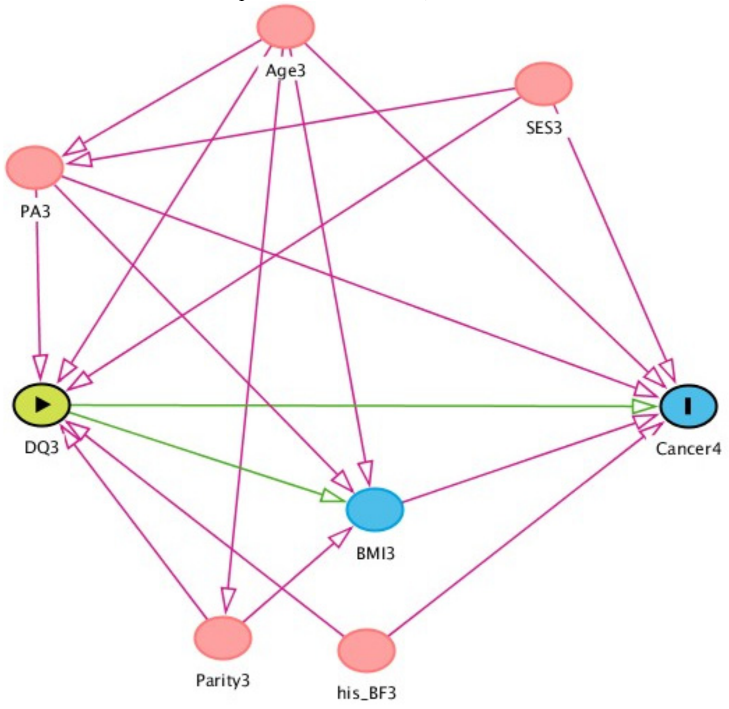

Supplementary Figure S4: A Directed Acyclic Graph (DAG) used to identify potential confounders for cancer at survey 4

BMI-Body mass index; DQ-diet quality; his_BF-history of breastfeeding; PA-physical activity; SES-socioeconomic status (residence, marital status, education, occupation, income stress)

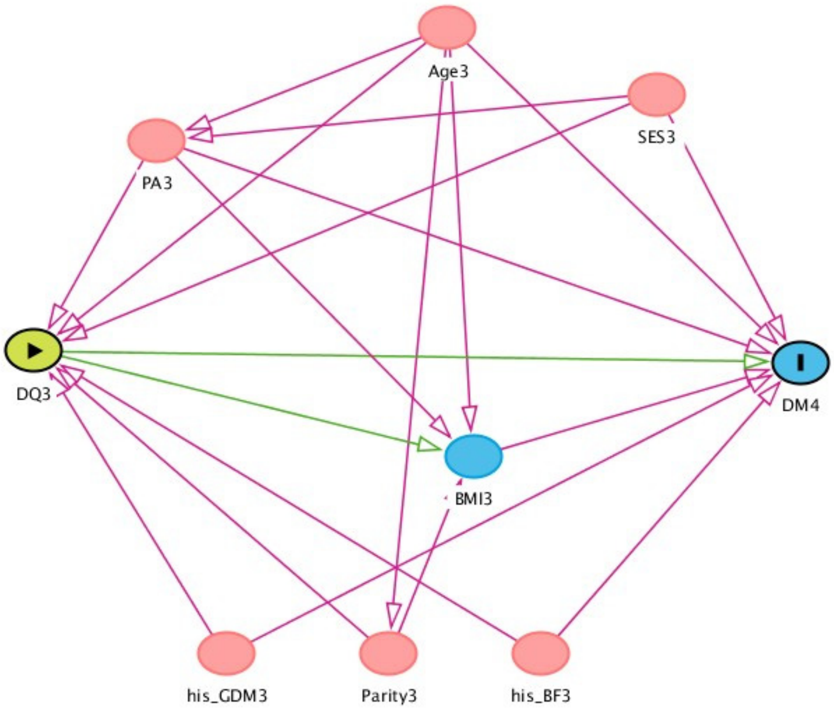

Supplementary Figure S5: A Directed Acyclic Graph (DAG) used to identify potential confounders for diabetes mellitus at survey 4

BMI-Body mass index; DM-diabetes mellitus; DQ-diet quality; his_BF-history of breastfeeding; his_GDM-history of gestational diabetes mellitus; PA-physical activity; SES-socioeconomic status (residence, marital status, education, occupation, income stress)

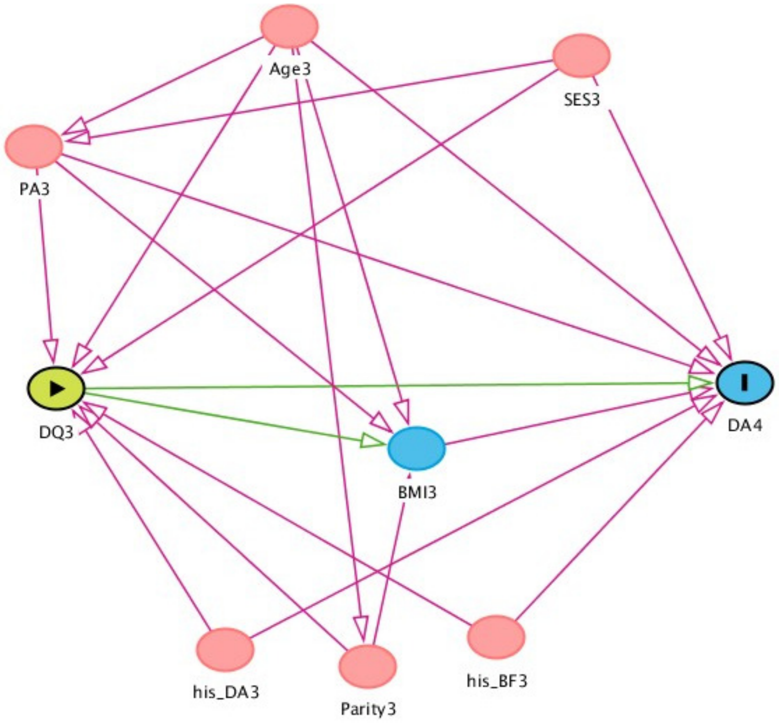

Supplementary Figure S6: A Directed Acyclic Graph (DAG) used to identify potential confounders for depression and/or anxiety at survey 4

BMI-Body mass index; DA-depression and/or anxiety; DQ-diet quality; his_BF-history of breastfeeding; his_DA-history of depression and/ or anxiety; PA-physical activity; SES-socioeconomic status (residence, marital status, education, occupation, income management)

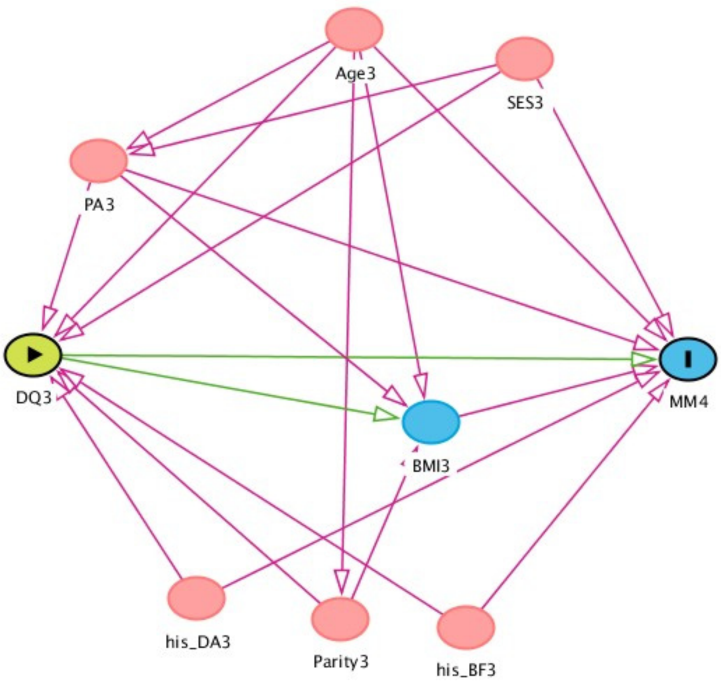

Supplementary Figure S7: A Directed Acyclic Graph (DAG) used to identify potential confounders for multimorbidity at survey 4

BMI-Body mass index; DQ-diet quality; his_BF-history of breastfeeding; his_DA-history of depression and/ or anxiety; MM-multimorbidity; PA-physical activity; SES-socioeconomic status (residence, marital status, education, occupation, income management)

Supplementary Table S1. Respondents in each survey, from survey 4 to survey 8, based on our sample

	**Survey 4**	**Survey 5**	**Survey 6**	**Survey 7**	**Survey 8**
Year	2006	2009	2012	2015	2018
Age survey age	26.6-34.6	39.6-37.3	32.8-40.6	35.7-43.4	40-46
Mean age	30.6 (1.5)	33.7 (1.5)	36.7 (1.5)	39.7 (1.5)	42.4 (1.5)
Deceased *	4	10	17	23	34
Non-respondents *	1,142	1,880	1,983	2,542	2,589
Respondents	6,871	6,127	6,017	5,452	5,394

** Numbers are cumulative*

Supplementary Table S2. Criteria for scoring Alternative Healthy Eating Index-2010

**Index component**	**Minimum (0 point)**	**Maximum (10 points)**
Vegetables per day	0 serve	≥ 5 serves
Fruits per day	0 serve	≥ 4 serves
Cereals (whole grain) per day	0 g	M: 90 g<br>F: 75 g
Legume and nuts per day	0 serve	≥ 1 serve
Red and processed meat per day	≥ 1.5 serves	0 serve
Trans-fat (% of energy)	≥ 4	≤ 0.5
Long-chain (n-3) fats (EPA+DHA) per day	0 mg	250 mg
PUFA (% of energy)	≤ 2	≥ 10
Sodium per day	Highest decile	Lowest decile
§ Alcohol consumption per day	Male: ≥ 3.5 drinks<br>Female: ≥ 2.5 drinks	Male: 0.5-2.0 drinks<br>Female: 0.5-1.5 drinks
Sugar-sweetened beverages and fruit juice per day	≥ 1 serve	0 serve

Intermediate amount scored proportionately. § Moderate alcohol consumption contributes to a higher Alternative Healthy Eating Index-2010 score (i.e higher diet quality). In this study, women who consumed alcohol 0.5-1.5 drinks were given the highest score "10"; 1.6-2.4 drinks were given the intermediate score "1-9"; ≥2.5 drinks were given the lowest score "0".

Supplementary Table S3. Cumulative incidence of NCDs, multimorbidity and mortality from Survey 4, S4 to Survey 8, S8 (N=8,017)

NCDs	Survey 4	Survey 5	Survey 6	Survey 7	Survey 8
	n (%)	n (%)	n (%)	n (%)	n (%)
**Coronary heart disease**					
CHD *	11 (0.1)	6 (0.1)	12 (0.2)	14 (0.2)	28 (0.4)
CHD [a]	11 (0.1)	17 (0.2)	29 (0.4)	42 (0.5)	69 (0.9)
**Hypertension**					
HT*	77 (1.0)	179 (2.2)	178 (2.2)	188 (2.4)	258 (3.2)
HT[a]	77 (1.0)	231 (2.9)	346 (4.3)	433 (5.4)	556 (6.9)
**Asthma**					
Asthma *	662 (8.3)	559 (7.0)	558 (7.0)	464 (5.8)	478 (6.0)
**Cancer**					
Cancer *	68 (0.9)	41 (0.5)	57 (0.7)	67 (0.8)	90 (1.1)
Cancer [a]	68 (0.9)	100 (1.3)	148 (1.9)	200 (2.5)	274 (3.4)
**Diabetes mellitus**					
DM *	24 (0.3)	45 (0.6)	54 (0.7)	44 (0.6)	57 (0.7)
DM [a]	24 (0.3)	62 (0.8)	110 (1.4)	136 (1.7)	167 (2.1)
**Depression and/or anxiety**					
Depression and/or anxiety *	999 (12.5)	1106 (13.8)	1199 (15.0)	1058 (13.2)	1024 (12.8)
**Multimorbidity**					
Multimorbidity [ł]	198 (2.5)	253 (3.2)	360 (4.5)	346 (4.3)	413 (5.2)
**All-cause mortality (between S4 and S8)**					
All-cause mortality	34 (0.4)				

Details about ALSWH surveys are available on the study website (http://www.alswh.org.au).
CHD: coronary heart disease; DM: diabetes mellitus; HT: hypertension.
Non-communicable diseases (except asthma, depression and/or anxiety) were considered as enduring condition. * Numbers in these rows were participants who had that disease at the time of data collection. [a] Numbers in these rows were participants who had that disease considered as enduring conditions. [ł] Multimorbidity was calculated by enduring condition of NCDs (except asthma, depression and/ or anxiety).

Supplementary Table S4. Comparison of socio-demographic and behavioural characteristics of participants those included and excluded in the study sample (at Survey 1, 1996)

Characteristics	Participants at Survey 1	Participants at Survey 3		P-value [a]
	N=14,247 [n (%)]	Included (n=8,017) [n (%)]	Excluded (n=1,064) [n (%)]	
Marital status				<0.001
Never married	10850 (76.6)	6229 (78.1)	741 (70.0)	
Married/de facto	3193 (22.5)	1707 (21.4)	297 (28.1)	
Separated/divorced/widowed	134 (0.9)	44 (0.5)	20 (1.9)	
Area of residence				0.04
Major cities	7375 (51.8)	4090 (51.1)	499 (46.9)	
Inner regional	4307 (30.3)	2488 (31.0)	353 (33.2)	
Outer regional/ rural	2555 (17.9)	1432 (17.9)	212 (19.9)	
Education				<0.001
No formal education	408 (2.9)	128 (1.6)	41 (3.9)	
High school level	9619 (67.9)	5382 (67.5)	697 (65.9)	
Diploma	2563 (18.1)	1412 (17.7)	223 (21.1)	
University degree	1576 (11.1)	1054 (13.2)	97 (9.1)	
Occupation				<0.001
No paid employment	1283 (9.2)	539 (6.8)	125 (12.0)	
Paid employment	12668 (90.8)	7362 (93.2)	917 (88.0)	

Income stress				<0.001
Easy	6865 (48.4)	4202 (52.6)	425 (40.1)	
Difficult	7330 (51.6)	3787 (47.4)	634 (59.9)	
Physical activity				0.03
None/ sedentary	895 (9.5)	617 (9.2)	89 (9.9)	
Low	3050 (32.3)	2157 (32.1)	326 (36.1)	
Moderate	2208 (23.4)	1627 (24.2)	186 (20.6)	
High	3283 (34.8)	2317 (34.5)	301 (33.4)	
Self-rated health				<0.001
Excellent	1773 (12.5)	1087 (13.6)	85 (8.0)	
Very good	5469 (38.6)	3289 (41.3)	355 (33.5)	
Good	5208 (36.8)	2767 (34.7)	434 (40.9)	
Fair/ poor	1716 (12.1)	827 (10.4)	186 (17.5)	
Taking prescribed medicine				0.002
No	6054 (42.6)	3431 (42.9)	402 (37.8)	
Yes	8149 (57.4)	4560 (57.1)	661 (62.2)	

Due to missing data, the sum for each characteristic may not equal n.
[a] Pvalues from chi-squared tests

Supplementary Table S5. Odds of common non-communicable diseases (including multimorbidity) over 15 years of follow-up (from survey 4, S4 to survey 8, S8) based on quintiles of Alternative Healthy Eating Index-2010: 1973-78 Australian Longitudinal Study on Women's Health cohort, by accounting for taking prescribed medicine.

NCD	OR (95% CI)	OR (95% CI)	OR (95% CI)	OR (95% CI)	OR (95% CI)
**HT**	***n = 77***	***n = 231***	***n = 346***	***n = 433***	***n = 556***
	S4 (n = 6,536)[b]	S5 (n = 5,845)[b]	S6 (n = 5,750)[b]	S7 (n = 5,219)[b]	S8 (n = 5,163)[b]
Multivariate [c]	1.1 (0.5-2.5)	0.8 (0.5-1.3)	0.7 (0.5-1.1)	0.7 (0.5-1.1)	0.8 (0.6-1.1)
**Asthma**	***n = 662***	***n = 559***	***n = 558***	***n = 464***	***n = 478***
	S4 (n = 6,549)[b]	S5 (n = 5,854)[b]	S6 (n = 5,760)[b]	S7 (n = 5,230)[b]	S8 (n = 5,175)[b]
Multivariate [d]	0.8 (0. 6-1.0)	0.8 (0.6-1.1)	0.9 (0.6-1.2)	0.8 (0.6-1.2)	0.8 (0.6-1.1)
Multivariate [d+]	0.8 (0.6-1.1)	0.8 (0.6-1.1)	0.9 (0.7-1.3)	0.8 (0.6-1.2)	0.8 (0.6-1.1)
**Cancer (excludes skin cancer)**	***n = 68***	***n = 100***	***n = 148***	***n = 200***	***n = 274***
	S4 (n = 6,549)[b]	S5 (n = 5,806)[b]	S6 (n = 5,721)[b]	S7 (n = 5,230)[b]	S8 (n = 5,175)[b]
Multivariate	1.4 (0.6-3.1)	1.4 (0.7-3.0)	1.1 (0.6-2.0)	0.9 (0.5-1.6)	0.9 (0.6-1.3)
**DM**	***n = 24***	***n = 62***	***n = 110***	***n = 136***	***n = 167***
	S4 (n = 6,488)[b]	S5 (n = 5,845)[b]	S6 (n = 5,750)[b]	S7 (n = 5,219)[b]	S8 (n = 5,163)[b]
Multivariate [e]	0.6 (0.1–3.4)	1.2 (0.4–3.8)	0.7 (0.3–1.4)	0.6 (0.3–1.4)	0.6 (0.3–1.1)
**Depression and/or anxiety**	***n = 999***	***n = 1,106***	***n = 1,199***	***n = 1,058***	***n = 1,024***
	S4 (n = 6,549)[b]	S5 (n = 5,854)[b]	S6 (n = 5,760)[b]	S7 (n = 5,230)[b]	S8 (n = 5,175)[b]
Multivariate [f]	1.1 (0.9-1.4)	1.0 (0.8-1.3)	1.1 (0.9-1.4)	1.0 (0.8-1.3)	1.0 (0.8-1.3)
**Multimorbidity**	***n = 198***	***n = 253***	***n = 360***	***n = 346***	***n = 413***
	S4 (n = 6,549)[b]	S5 (n = 5,854)[b]	S6 (n = 5,760)[b]	S7 (n = 5,230)[b]	S8 (n = 5,175)[b]
Multivariate [f]	1.2 (0.7-2.0)	0.9 (0.6-1.5)	0.9 (0.6-1.3)	1.0 (0.7-1.5)	0.8 (0.6-1.2)

CHD: coronary heart disease; CI: confidence interval; DM: diabetes mellitus; HT: hypertension; NCD: non-communicable disease; OR: odds ratio; S: survey. OR (95% CI) expressed in the table is the comparison of odds of having NCDs (each disease and multimorbidity) in the highest quintile to the lowest quintile of AHEI-2010. Age, socioeconomic status (marital status, residence, education, occupation, and income stress), the behavioural variable (physical activity), and taking prescribed medicine were adjusted for in multivariate models of all NCD outcomes. [b] Number in bracket shows the number of participants in respective surveys for multivariate regression; [c] A history of hypertension during pregnancy at S3 was included as a covariate; [d] Model without a history of asthma at S3; [d+] Model with a history of asthma at S3; [e] A history of gestational diabetes mellitus at S3 was included as a covariate; [f] A history of depression and/or anxiety at S3 was included as a covariate; Texts in bold and italic represent accumulative figures of NCD cases (except asthma, depression and/or anxiety) in every survey.

Supplementary Table S6. Odds of common non-communicable diseases (including multimorbidity) over 15 years of follow-up (from survey 4, S4 to survey 8, S8) based on quintiles of Alternative Healthy Eating Index-2010: 1973-78 Australian Longitudinal Study on Women's Health cohort, by accounting for changes of childbearing variables (current pregnancy, parity, breastfeeding, history of gestational diabetes mellitus, history of hypertension during pregnancy).

NCD	OR (95% CI)	OR (95% CI)	OR (95% CI)	OR (95% CI)	OR (95% CI)
**HT**	***n = 77***	***n = 231***	***n = 346***	***n = 433***	***n = 556***
	S4 (n = 6,574)[b]	S5 (n = 5,862)[b]	S6 (n = 5,747)[b]	S7 (n = 5,032)[b]	S8 (n = 4,860)[b]
Multivariate 1[c]	1.1 (0.5-2.3)	0.7 (0.4-1.2)	0.7 (0.5-1.1)	0.9 (0.6-1.4)	0.9 (0.6-1.3)
	S4 (n = 6,502)[b]	S5 (n = 5,802)[b]	S6 (n = 5,684)[b]	S7 (n = 4,987)[b]	S8 (n = 4,814)[b]
Multivariate 2[c]	1.1 (0.5-2.5)	0.8 (0.5-1.3)	0.8 (0.5-1.2)	0.9 (0.6-1.4)	0.9 (0.6-1.3)
**Asthma**	***n = 662***	***n = 559***	***n = 558***	***n = 464***	***n = 478***
	S4 (n = 6,597)[b]	S5 (n = 5,909)[b]	S6 (n = 5,798)[b]	S7 (n = 5,083)[b]	S8 (n = 5,071)[b]
Multivariate 1[d]	0.8 (0.6-1.0)	0.8 (0.6-1.0)	0.9 (0.6-1.2)	0.8 (0.6-1.2)	0.8 (0.6-1.1)
	S4 (n = 6,525)[b]	S5 (n = 5,849)[b]	S6 (n = 5,735)[b]	S7 (n = 5,037)[b]	S8 (n = 5,023)[b]
Multivariate 2[d]	0.8 (0.6-1.0)	0.8 (0.6-1.1)	0.9 (0.7-1.2)	0.9 (0.6-1.2)	0.8 (0.6-1.1)
	S4 (n = 6,597)[b]	S5 (n = 5,909)[b]	S6 (n = 5,798)[b]	S7 (n = 5,083)[b]	S8 (n = 5,071)[b]
Multivariate 1[d+]	0.8 (0.6-1.1)	0.8 (0.6-1.1)	1.0 (0.7-1.3)	0.9 (0.6-1.3)	0.8 (0.6-1.2)
	S4 (n = 6,525)[b]	S5 (n = 5,849)[b]	S6 (n = 5,735)[b]	S7 (n = 5,037)[b]	S8 (n = 5,023)[b]
Multivariate 2[d+]	0.8 (0.6-1.1)	0.8 (0.6-1.1)	1.0 (0.7-1.3)	0.9 (0.6-1.3)	0.8 (0.6-1.2)
**Cancer (excludes skin cancer)**	***n = 68***	***n = 100***	***n = 148***	***n = 200***	***n = 274***
	S4 (n = 6,597)[b]	S5 (n = 5,861)[b]	S6 (n = 5,760)[b]	S7 (n = 5,083)[b]	S8 (n = 5,071)[b]
Multivariate 1	1.3 (0.6-3.1)	1.4 (0.7-3.0)	1.1 (0.6-2.0)	1.1 (0.6-1.9)	0.9 (0.6-1.3)
	S4 (n = 6,525)[b]	S5 (n = 5,801)[b]	S6 (n = 5,697)[b]	S7 (n = 5,037)[b]	S8 (n = 5,023)[b]
Multivariate 2	1.3 (0.6-3.1)	1.4 (0.7-2.9)	1.1 (0.6-2.0)	1.1 (0.6-2.0)	0.9 (0.6-1.3)
**DM**	***n = 24***	***n = 62***	***n = 110***	***n = 136***	***n = 167***
	S4 (n = 6,526)[b]	S5 (n = 5,863)[b]	S6 (n = 5,748)[b]	S7 (n = 5,029)[b]	S8 (n = 4,857)[b]
Multivariate 1[e]	0.9 (0.2–4.0)	1.8 (0.6–5.4)	0.7 (0.3–1.5)	0.8 (0.4–1.8)	0.6 (0.3–1.2)
	S4 (n = 6,454)[b]	S5 (n = 5,804)[b]	S6 (n = 5,686)[b]	S7 (n = 4,985)[b]	S8 (n = 4,812)[b]
Multivariate 2[e]	0.6 (0.1-3.3)	1.5 (0.5-4.7)	0.6 (0.3-1.4)	0.8 (0.4-1.7)	0.6 (0.3-1.2)
**Depression and/or anxiety**	***n = 999***	***n = 1,106***	***n = 1,199***	***n = 1,058***	***n = 1,024***
	S4 (n = 6,597)[b]	S5 (n = 5,909)[b]	S6 (n = 5,798)[b]	S7 (n = 5,083)[b]	S8 (n = 5,071)[b]
Multivariate 1[f]	1.0 (0.8-1.3)	0.9 (0.7-1.2)	1.2 (0.9-1.5)	1.1 (0.8-1.4)	1.0 (0.8-1.3)
	S4 (n = 6,525)[b]	S5 (n = 5,849)[b]	S6 (n = 5,735)[b]	S7 (n = 5,037)[b]	S8 (n = 5,023)[b]
Multivariate 2[f]	1.0 (0.8-1.3)	0.9 (0.7-1.2)	1.2 (0.9-1.5)	1.1 (0.8-1.4)	1.0 (0.8-1.3)
**Multimorbidity**	***n = 198***	***n = 253***	***n = 360***	***n = 346***	***n = 413***
	S4 (n = 6,597)[b]	S5 (n = 5,909)[b]	S6 (n = 5,798)[b]	S7 (n = 5,083)[b]	S8 (n = 5,071)[b]
Multivariate 1[f]	1.1 (0.7-1.8)	0.9 (0.5-1.4)	0.9 (0.6-1.3)	1.1 (0.7-1.6)	0.9 (0.6-1.3)
	S4 (n = 6,525)[b]	S5 (n = 5,849)[b]	S6 (n = 5,735)[b]	S7 (n = 5,037)[b]	S8 (n = 5,023)[b]
Multivariate 2[f]	1.2 (0.7-1.9)	0.9 (0.6-1.4)	0.9 (0.6-1.4)	1.1 (0.7-1.7)	0.9 (0.6-1.2)

CHD: coronary heart disease; CI: confidence interval; DM: diabetes mellitus; HT: hypertension; NCD: non-communicable disease; OR: odds ratio; S: survey. OR (95% CI) expressed in the table is the comparison of odds of having NCDs (each disease, multimorbidity) in the highest quintile to the lowest quintile of AHEI-2010. Multivariate 1: model without prescribed medicine; Multivariate 2: model with prescribed medicine. Age, socioeconomic status (marital status, residence, education, occupation, and income stress), the behavioural variable (physical activity), and taking prescribed medicine were adjusted for in multivariate models of all NCD outcomes. [b] Number in bracket shows the number of participants in respective surveys for multivariate regression; [c] A history of hypertension during pregnancy at S3 was included as a covariate; [d] Model without a history of asthma at S3; [d+] Model with a history of asthma at S3; [e] A history of gestational diabetes mellitus at S3 was included as a covariate; [f] A history of depression and/or anxiety at S3 was included as a covariate; Texts in bold and italic represent accumulative figures of NCD cases (except asthma, depression and/or anxiety) in every survey.
